# Mendelian Randomization and Machine Learning Reveal Immune Cell and Gene Drivers in Systemic Lupus Erythematosus

**DOI:** 10.1002/brb3.70754

**Published:** 2025-09-16

**Authors:** Luofei Huang, Jian shi, Han Li, Quanzhi Lin

**Affiliations:** ^1^ Liuzhou Municipal Liutie Central Hospital Liuzhou Guangxi China; ^2^ Department of Internal Medicine The People's Hospital of Laibin Laibin Guangxi China; ^3^ Liuzhou People's Hospital Affiliated to Guangxi Medical University Liuzhou Guangxi China; ^4^ The First Affiliated Hospital of Guangxi University of Science and Technology Liuzhou Guangxi China

**Keywords:** biomarkers, immune cell phenotypes, machine learning, Mendelian randomization, systemic lupus erythematosus

## Abstract

**Background:**

Systemic lupus erythematosus (SLE) is a complex autoimmune disease with unclear pathogenesis. Recent studies suggest that immune cell phenotypes may play a causal role. This study aimed to uncover causal immune cell types, key genes, and potential biomarkers using Mendelian randomization (MR) and machine learning.

**Methods:**

A two‐sample MR analysis was performed on 731 immune cell traits to assess their causal relationship with SLE risk. Gene Expression Omnibus datasets were used to identify differentially expressed genes (DEGs), followed by immune infiltration analysis and machine learning‐based gene selection. Key genes were validated using independent datasets and expression quantitative trait loci‐MR analysis.

**Results:**

Significant causal links with SLE were observed for 10 immune cell subtypes (*p* < 0.01). A total of 17 DEGs, including FCGR2A, TMEM181, and RASA3, were identified as being associated with immune infiltration. Single‐sample gene set enrichment analysis revealed altered immune cell compositions in SLE. Five key genes (FCGR2A, TMEM181, RASA3, BCAR3, and MCTP2) with strong diagnostic potential (area under the curve = 0.948) were identified using a support vector machine model. Their causal relevance was confirmed by MR.

**Conclusions:**

This integrative approach revealed 10 immune cell types and five genes with causal roles in SLE, offering novel insights into disease mechanisms and potential targets for precision medicine.

## Graphical abstract

Flow schematic diagram

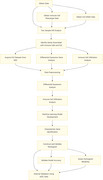

## 1 Introduction

Systemic lupus erythematosus (SLE) is a multifactorial autoimmune disease characterized by abnormal immune activation and chronic inflammation, resulting in multisystem involvement (Kiriakidou and Ching 2020; Mills 1994). Epidemiological studies reveal a female predominance (particularly in childbearing age) and a higher prevalence in non‐European populations (Jiménez et al. 2003; Siegel and Lee 1973). Despite progress in understanding its pathogenesis, the precise mechanisms driving the onset of the disease remain unclear (Ichinose et al. 2021; Kato and Kahlenberg 2024). Current therapies, including immunosuppressants and B cell and cytokine‐targeted biologics, are limited by side effects, variable efficacy, and risks of long‐term complications (for instance, infection and organ damage), highlighting the need for mechanistic insights and precision treatments.

A central component of SLE pathophysiology is the interplay between immune cell phenotypes and the disease (Accapezzato et al. 2023). T cells, B cells, and monocytes contribute to SLE progression through their dysregulated responses and cytokine production, which initiate and maintain inflammation (Möckel et al. 2021; Herrada et al. 2019). Determining the precise causative roles of immune cell subtypes in SLE can help identify novel therapeutic targets and personalized strategies, particularly regarding how these subtypes interact with autoantigens and affect disease activity.

Mendelian randomization (MR) has been extensively used in medical studies (Liu 2023; Liu et al. 2023). It provides a robust framework for inferring causality by using genetic variants as instrumental variables, thereby mitigating confounding and reverse causation biases in observational studies (Barrett et al. 2013). Integrating MR with transcriptomic data from the Gene Expression Omnibus (GEO) enables the determination of changes in gene expression that correlate with and potentially contribute to SLE risk (Gupta et al. 2017; Didelez and Sheehan 2007). This approach facilitates the identification of genetic factors and molecular pathways, bridging genetic predispositions to pathological processes. Large‐scale GEO datasets enable comprehensive analysis of gene expression profiles, facilitating the identification of biomarkers and the development of treatments. This study combined MR, transcriptomic analysis, and machine learning to elucidate the causal associations between immune cells and SLE and identify key pathogenic genes.

## 2 Materials and Methods

### 2.1 Study Design

A multi‐step analytical framework was used in this study to elucidate the phenotypes of immune cells and essential genes associated with SLE. Initially, a two‐sample MR analysis was conducted to assess the causal relationships between 731 immune cell phenotypes and SLE risk, aiming to identify genes that are simultaneously associated with immune cell exposure and the development of SLE. Subsequently, three sets of SLE transcriptomic datasets, comprising two training sets and one validation set, were integrated from the GEO database. These datasets were processed through several steps, including background correction, batch effect removal using the surrogate variable analysis algorithm, and differentially expressed genes (DEGs) analysis (Leek et al. 2012). Characteristic genes were filtered out in conjunction with immune infiltration analysis and machine learning models. Finally, the causal impacts of gene expression on SLE were conclusively validated using an independent dataset and MR analysis of expression quantitative trait loci (eQTL).

### 2.2 Data Sources

The data on immune cell phenotypes were sourced from the Genome‐Wide Association Studies (GWAS) conducted by the Blood Cell Consortium, which included 731 blood cell traits of 563,085 participants with European ancestry. These traits comprehensively covered various cell types, including leukocytes, monocytes, lymphocytes, and their subtypes, such as HLA DR+ natural killer (NK) cells and CD4+ regulatory T cells. The GWAS summary data for SLE comprised 7,071,163 single‐nucleotide polymorphisms (SNPs) from 14,267 samples, including 5201 patients and 9066 controls. Regarding the datasets from the GEO database, GSE50772 and GSE81622 served as the training sets, while GSE61635 functioned as the validation set. These datasets represented transcriptomic data of human peripheral blood samples, as detailed in Table S1.

### 2.3 MR Analysis

A two‐sample MR analysis was performed using the TwoSampleMR package to investigate the causal relationship between 731 immune cell phenotypes and the risk of SLE (Zoccali 2017). The primary MR method employed was inverse variance weighting (IVW) with random effects (Carter et al. 2022). When a single SNP was available as the instrument for exposure, the Wald ratio method was applied instead. The MR package (version 0.4.3) was used, and additional analyses were conducted using the weighted median and IVW methods to ensure the robustness of MR results (Higbee et al. 2021). Residual heterogeneity was assessed using Cochran's *Q* test, and a significance threshold of *p* < 0.05 indicated the presence of heterogeneity (Zhao et al. 2023). Potential horizontal pleiotropy was evaluated using the MR‐Egger intercept test, where *p* < 0.05 indicated the existence of horizontal pleiotropy (Fang et al. 2023).

### 2.4 Identification and Analysis of DEGs

Gene expression data from control and SLE groups were obtained from the integrated GEO dataset. According to previous studies, genes with a *p* value less than 0.05 were identified as differentially expressed SLE associated genes (DEAGs) (Y. Li and Liu 2022; Liu and Li 2022; Liu and Weng 2022b). The results of the differential expression analysis were illustrated using boxplots. Furthermore, the chromosomal distribution of DEAGs was examined, and their distribution patterns were visually represented. Pairwise correlation coefficients among all DEAGs were also calculated, and the analysis outcomes were displayed through graphical visualization.

### 2.5 Investigation of Immune Cell Profiles in SLE Samples

Single‐sample gene set enrichment analysis (ssGSEA) was used to compare the differences in the composition of immune cells between the control and SLE groups, and the results are presented as boxplots (Subramanian et al. 2005). DEAGs were then matched to ssGSEA scores, and correlation analyses were performed to calculate the correlation coefficients. The findings are visualized using heatmaps.

### 2.6 Development and Validation of Predictive Models and Nomograms for Identifying Characteristic Genes in SLE

A total of nine predictive models were developed using the expression data of DEAGs, including least absolute shrinkage and selection operator (Kang et al. 2021; Liu and Tang 2023b; Liu and Tang 2023a), neural network, extreme gradient boosting, gradient boosting machine, support vector machine, random forest (Hu and Szymczak 2023), K‐nearest neighbors, decision tree, and generalized linear model (Y. Wang et al. 2022). After calculating the prediction functions, the most suitable model was determined by analyzing residual boxplots and receiver operating characteristic (ROC) curves (Liu and Weng 2022a; Liu, Karsidag, et al. 2025). Characteristic genes were selected from the DEAGs using this method. A nomogram was constructed based on the expression levels of these characteristic genes in the control and SLE groups. Decision curves and calibration plots were generated to evaluate the accuracy and generalizability of the nomogram. The reliability of the training model was then confirmed by plotting the ROC curve after the same model was built using the validation dataset. MR analyses were conducted using the eQTL data of the characteristic genes for external validation. The causal effects of gene and protein expression levels on SLE were assessed using these analyses.

## 3 Results

### 3.1 MR Analysis

**FIGURE 1:**
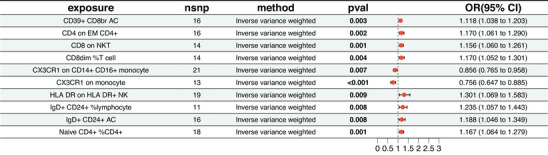
Forest plot of the MR analysis between immune cell phenotypes and SLE; IVW, inverse variance weighted; OR, odds ratio.

This study identified a potential causal relationship between 10 types of immune cells and the risk of SLE (*p* < 0.01). Specifically, higher levels of CD39+ CD8br AC, CD8 on NKT, CD8dim %T cells, HLA‐DR on HLA‐DR+ NK, IgD+ CD24+ %lymphocytes, IgD+ CD24+ AC, and naïve CD4+ %CD4+ cells were associated with an increased risk of SLE. Conversely, higher levels of CX3CR1 on CD14+ CD16+ monocytes and CX3CR1 expression on monocytes were linked to a decreased risk of SLE (Figure 1). Finally, this analysis revealed no evidence of pleiotropy (Table S2) or significant heterogeneity (Table S3).

### 3.2 Identification and Analysis of DEAGs

**FIGURE 2:**
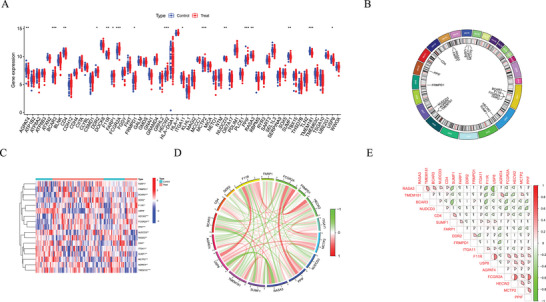
Identification and analysis of DEAGs in SLE. (A) Boxplots illustrating the expression differences of relevant genes between the control and SLE groups. (B) Circos plot demonstrating the chromosomal locations of DEAGs. (C) Heatmap of the expression levels of DEAGs. (D) Network diagram of DEAG correlations. (E) Heatmap analyzing the correlations among DEAGs.

This analysis also identified 85 genes associated with both immune cell phenotypes and SLE risk (Table S4). The expression profiles of these genes in the control and SLE groups are illustrated in Figure 2A. Among these, 17 were identified as differentially expressed SLE associated genes (DEAGs), including AGPAT4, BCAR3, CD4, DDR2, F11R, FARP1, FCGR2A, FRMPD1, HECW2, ITGA11, MCTP2, NUDCD3, PPIF, RASA3, SUMF1, TMEM181, and USP8 (Table S6). Their chromosomal locations are mapped in Figure 2B, while their expression patterns across samples are presented in a heatmap (Figure 2C). Correlation analysis among DEAGs in SLE samples revealed predominantly positive associations (Figure 2D); their interrelationships are further visualized in a network diagram (Figure 2E).

### 3.3 Immune Cell‐Related Analysis

**FIGURE 3:**
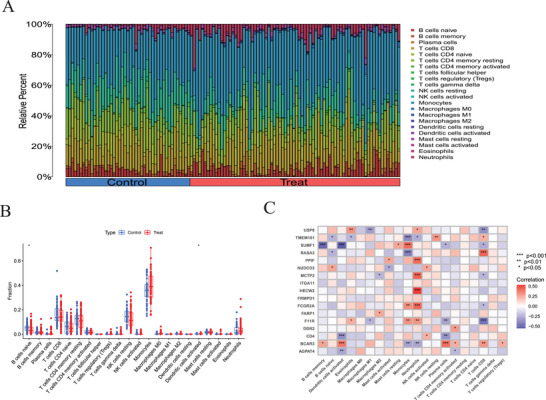
Analysis related to immune cell infiltration. (A) Bar graph depicting the relative percentages of each immune cell type within the samples. (B) Boxplots comparing the immune cell scores between the control and SLE groups. (C) Heatmap of the correlation analysis between DEAGs and immune cell types.

The types and proportions of immune cells in each sample were quantified by the immune cell infiltration analysis (Figure 3A). Two immune cell types exhibited significant differences between the control and SLE groups, as determined by ssGSEA (Figure 3B): the control group had a higher abundance of naïve B cells and activated dendritic cells. In addition, correlation analysis demonstrated primarily negative associations between differentially expressed SLE associated genes (DEAGs) and immune cell populations. Correlations between DEAGs are also displayed to provide further insights into their potential regulatory relationships (Figure 3C).

### 3.4 Machine Learning Model Selection, Nomogram Construction, and Validation Results

**FIGURE 4:**
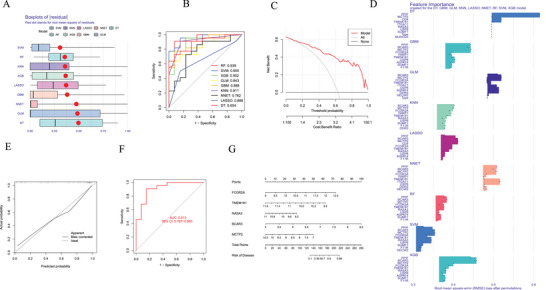
Machine learning analysis and nomogram construction and validation based on DEAGs. (A) Residual boxplots for nine machine learning models. (B) ROC curves for the training dataset across nine machine learning models. (C) Decision curves of the nomogram for characteristic genes. (D) Feature importance bar graphs for the nine machine learning models. (E) Calibration curves. (F) ROC curve for validating gene expression composites on the GEO dataset. (G) Nomogram for characteristic genes. DT, decision tree; GBM, gradient boosting machine; GLM, generalized linear model; KNN, k‐nearest neighbors; LASSO, least absolute shrinkage and selection operator; NNET, neural network; RF, random forest; SVM, support vector machine; XGB, extreme gradient boosting.

**FIGURE 5:**
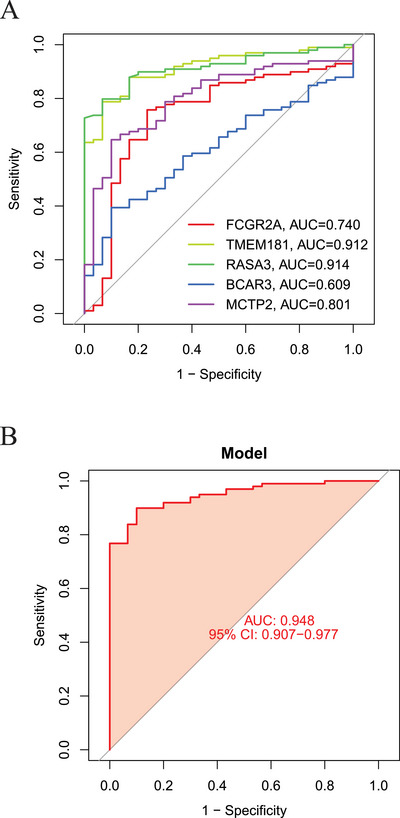
External validation of characteristic genes and nomogram model accuracy. (A) ROC curves of five characteristic genes in the GEO61635 validation cohort. (B) ROC curve of the nomogram model integrating five genes of external validation.

Figures 4A,B demonstrate that the SVM method exhibited the largest area under the ROC curve and the smallest residuals among the nine machine‐learning models constructed in this study. Consequently, SVM was selected for further analysis to determine the importance scores of characteristic genes (Figure 4D). The top five genes with the highest importance scores that were used to construct the nomogram were FCGR2A, TMEM181, RASA3, BCAR3, and MCTP2. Scoring scales for these five characteristic genes were then established individually. The risk rates associated with the characteristic genes were evaluated by adding up the scores of the characteristic gene expressions (Figure 4G). The accuracy of the model was reflected by the distance between the red and gray lines on the decision curve (Figure 4C). Calibration plots demonstrated strong consistency between the predictions of the nomogram and actual outcomes, highlighting its reliability (Figure 4E). The generalized linear model was further validated using the validation dataset, with an area under the curve (AUC) of 0.913 (95% CI: 0.787−0.993), indicating excellent accuracy for the model based on the GEO dataset (Figure 4F). In addition, MR analysis was conducted using QTLs for external validation of the characteristic genes, and the results are provided in Table S5. Moreover, validation using the GEO61635 dataset revealed the following AUC values for the characteristic genes: FCGR2A (AUC = 0.740), TMEM181 (AUC = 0.912), RASA3 (AUC = 0.914), BCAR3 (AUC = 0.609), and MCTP2 (AUC = 0.801) (Figure 5A). The nomogram model based on these five common characteristic genes demonstrated high accuracy, with an AUC of 0.948 (95% CI: 0.907–0.977) (Figure 5B). These findings highlight the potential utility of these characteristic genes as biomarkers for the identification and diagnosis of SLE.

## 4 Discussion

This study integrated two‐sample MR, gene expression analysis, and machine learning to explore causal links between 731 immune cell phenotypes and SLE risk. Ten immune cell types were identified as being significantly associated with SLE, with some conferring protection and others increasing susceptibility. Simultaneously, machine learning and gene expression profiling of SLE datasets from GEO revealed five key feature genes: FCGR2A, TMEM181, RASA3, BCAR3, and MCTP2. These genes, which were validated in an independent dataset, suggest their potential roles in SLE pathogenesis. This discussion further explores the implications of these findings for understanding disease mechanisms and advancing therapeutic strategies. These genes, which were validated in an independent dataset, suggest their potential roles in SLE pathogenesis, including neuropsychiatric manifestations. This discussion further explores the implications of these findings for understanding disease mechanisms, particularly neuropsychiatric involvement, and advancing therapeutic strategies.

First, the observed associations between specific immune cell subtypes and SLE risk highlight the crucial role of immune dysregulation in disease development (Nehar‐Belaid et al. 2020). Specifically, increased levels of CD39+ CD8br AC, CD8 expression in NKT cells, CD8dim%T cells, HLA DR+ expression in NK cells, IgD+ CD24+ %lymphocytes, IgD+ CD24+ AC, and naïve CD4+ %CD4+ cells were significantly associated with a higher risk of SLE. These findings are consistent with the existing literature on the roles of T and NK cells in the pathophysiology of SLE (Sharabi and Tsokos 2020; Kucuksezer et al. 2021). For instance, the cytotoxic activity of CD8+ T cells may cause autologous tissue damage (Xiong et al. 2023). In contrast, abnormalities in NK cell function may hinder the elimination of apoptotic cells, thereby exposing autoantigens and perpetuating immune responses (Henriques et al. 2013). Moreover, high expression of CX3CR1 in CD14+ CD16+ monocytes is correlated with a reduced risk of SLE, indicating a protective role in maintaining immune homeostasis and suppressing inflammatory responses (Thomas et al. 2015). These findings broaden our understanding of the role of immune cells in SLE pathogenesis and emphasize potential therapeutic targets within specific immune cell subpopulations.

A total of 17 differentially expressed SLE associated genes (DEAGs) were identified by integrating three GEO datasets linked with SLE (GSE50772, GSE81622, and GSE61635). These include AGPAT4, BCAR3, CD4, DDR2, F11R, FARP1, FCGR2A, FRMPD1, HECW2, ITGA11, MCTP2, NUDCD3, PPIF, RASA3, SUMF1, TMEM181, and USP8. These genes exhibited significant differences in expression between SLE patients and controls, suggesting their involvement in disease onset. In addition, five key feature genes—FCGR2A, TMEM181, RASA3, BCAR3, and MCTP2—were found using machine learning models. These genes exhibited strong predictive performance (AUC = 0.948) in an independent validation set, confirming their potential as diagnostic and prognostic biomarkers in SLE.

Particularly, FCGR2A, a type of Fcγ receptor, plays a significant role in antibody‐dependent phagocytosis (Nagelkerke et al. 2019). Its increased expression in SLE may promote the formation and clearance of immune complexes, thereby exacerbating inflammatory responses and tissue damage (Tsang‐A‐Sjoe et al. 2016; Vyse and Kotzin 1998). Although the specific functions of TMEM181 and RASA3 in SLE are not entirely understood, their potential contributions to immune regulation and signaling suggest that they may be involved in modulating the activity and migration of immune cells. BCAR3 and MCTP2 are crucial in cell adhesion and membrane transport, respectively, and can affect the interactions and localization of immune cells, thereby playing roles in the immunopathology of SLE (Joshi et al. 2018). The expression of these genes was further validated for their causal impact on SLE risk through MR analysis, enhancing their credibility as potential biomarkers or therapeutic targets. Notably, neuropsychiatric symptoms (e.g., cognitive dysfunction, depression) are prevalent in SLE patients and may stem from immune‐mediated neuronal damage. The identified genes, particularly FCGR2A and RASA3, have been implicated in neuroinflammatory pathways. FCGR2A‐mediated phagocytosis of synaptic components by microglia could contribute to cognitive impairment, while RASA3's role in endothelial barrier integrity may influence blood‐brain barrier disruption in SLE encephalopathy. Future studies should explore these genes' expression in cerebrospinal fluid or brain tissue to clarify their neuropathological roles.

The ssGSEA in this study demonstrated significant differences in immune cell infiltration between SLE and control samples, with higher levels of naïve B cells and activated dendritic cells in the controls, indicating that SLE‐related inflammation may be associated with a decrease in regulatory or tolerant immune populations (Yang et al. 2020). In addition, a predominantly negative correlation was observed between DEAGs and immune cell content, further highlighting the complex interactions between gene expression and immune cell functionality in SLE. Moreover, this study revealed how data‐driven approaches may be used for screening and predicting disease‐characteristic genes by constructing and validating machine learning models. The SVM model performed best according to this analysis, with high predictive accuracy and stability. This outcome highlights the importance of selecting suitable machine learning algorithms for biomedical research and serves as a reference for future development of disease prediction models based on gene expression data. However, the quality and diversity of the training data significantly impact the performance of machine learning models, suggesting that introducing more diverse and high‐quality datasets may further enhance the generalizability and practicality of the models. The findings of this study have significant clinical implications for the diagnosis and treatment of SLE. The identified critical feature genes serve as potential biomarkers for early diagnosis, risk assessment, and monitoring treatment efficacy, and may also develop into novel therapeutic targets, thereby promoting personalized medicine. For instance, inhibitors targeting FCGR2A can help reduce the formation and deposition of immune complexes (Lood et al. 2017; Melki et al. 2020), thereby alleviating inflammatory responses and tissue damage.

Further research into the functions of TMEM181, RASA3, BCAR3, and MCTP2 in immune cells may elucidate the molecular pathways and regulatory networks underlying SLE pathophysiology, thereby providing a theoretical foundation for the development of new immunomodulatory therapies. Notably, it is becoming more widely accepted that immune cell dysfunction and dysregulated gene expression are the causes of neuropsychiatric symptoms of SLE, such as lupus encephalopathy and depression. The key genes identified in this study are implicated in immune‐inflammatory processes; therefore, their potential involvement in neuroimmune interactions warrants further exploration, especially as this area has gained prominence in recent research. Future studies should validate the expression and function of these genes across different SLE subtypes using both in vitro and in vivo models. High‐resolution information about their regulatory dynamics and roles specific to particular cell types may be obtained by incorporating single‐cell sequencing. In addition, the neuropsychiatric manifestations of SLE (e.g., psychosis and seizures) remain poorly understood mechanistically. The immune cell subtypes and key genes identified here, such as CX3CR1+ monocytes (which patrol the vasculature and modulate neuroinflammation) and MCTP2 (involved in calcium signaling in neurons), may offer new avenues to study SLE‐associated brain dysfunction. Single‐cell sequencing of peripheral and central nervous system samples could reveal cell‐type‐specific dysregulation of these targets. Moreover, expanding research across diverse populations with varied genetic backgrounds will be essential to confirm the robustness and global relevance of these findings. One of the notable strengths of this study is its comprehensive methodology, which combines large‐scale GWAS data, gene expression data, and advanced machine learning techniques to provide a multi‐level, multi‐dimensional analysis framework. This integrated approach enhances the reliability and repeatability of the research findings while also offering new insights for studying complex diseases such as SLE. Future studies can further investigate the roles of these genes in the development of neuropsychiatric symptoms, providing new directions for mechanistic studies on SLE‐related brain dysfunction. In addition, naturally derived drugs have demonstrated therapeutic potential across various diseases (Q. Li et al. 2025), including bone diseases (C. Wang et al. 2016), cancer (Liu 2022; Hengrui 2023; Liu 2020), brain disorders (Xu et al. 2024), liver diseases (Liu 2025), infections (Ou et al. 2024; Peng et al. 2023), and age‐related diseases (H. Wang et al. 2020). Future research should investigate how these compounds may regulate the phenotypes of immune cells and key genes in SLE.

### 4.1 Strengths and Limitations

This study also has certain limitations. First, GWAS data used primarily originates from European populations, so the findings may not be as generalizable to other races or populations. Differences in genetic backgrounds among populations can influence the association between immune cell phenotypes and disease risk; therefore, future research should encompass a broader range of populations to validate the universality of these findings. Second, although MR analysis reduces the impact of confounding factors to some extent, it is unable to eliminate the interference of pleiotropy or unmeasured confounding variables. Moreover, there are intrinsic limitations to open data (Liu, Li, et al. 2025; Liu et al. 2024). Accordingly, further functional experiments and mechanistic studies are still necessary to elucidate the specific roles and mechanisms of these important genes in SLE.

## 5 Conclusions

This study not only advances the understanding of systemic SLE pathogenesis but also provides candidate mechanisms for its neuropsychiatric manifestations. The causal involvement of immune cells like HLA‐DR+ NK cells and genes such as FCGR2A aligns with emerging evidence of neuro‐immune crosstalk in SLE‐related depression and encephalopathy. In conclusion, this study systematically integrates a variety of data sources and analytical approaches to reveal causal links between immune cell phenotypes and SLE risk while identifying key feature genes involved in the pathogenesis of the disease. Despite certain limitations, these findings offer valuable insights into the underlying mechanisms of SLE and highlight potential targets for future diagnosis, prevention, and therapeutic interventions with significant scientific and clinical implications.

## Author Contributions

**Luofei Huang**: writing – original draft, visualization. **Jian Shi**: conceptualization, methodology, validation, writing – review and editing. **Han Li**: conceptualization, visualization, software, writing – review and editing. **Quanzhi Lin**: funding acquisition, writing – review and editing, visualization, validation, methodology, software.

## Ethics Statement

The study was a public database study, so it did not require informed consent and ethical approval from the committee. All authors agreed to the publication of the paper.

## Conflicts of Interest

The authors declare no conflicts of interest.

## Peer Review

The peer review history for this article is available at https://publons.com/publon/10.1002/brb3.70754.

## Supporting information




**Supporting Table 1**: SLE‐related microarray datasets.

**Supporting Table 2**: Table of Horizontal Pleiotropy Test Results in the MR Analysis of Immune Cell Phenotypes and Systemic Lupus Erythematosus.

**Supporting Table 3**: Table of Heterogeneity Test Results in the MR Analysis of Immune Cell Phenotypes and Systemic Lupus Erythematosus.

**Supporting Table 4**: Table of Genes Associated with Both Immune Cell Phenotypes and SLE Risk

**Supporting Table 5**: Table of Mendelian Randomization Analysis Results for Expression Quantitative Trait Loci of Characteristic Genes.

**Supporting Table 6**:| Gene name conversion.

## Data Availability

The data that support the findings of this study are available on request from the corresponding author. The data are not publicly available due to privacy or ethical restrictions.

## References

[brb370754-bib-0001] Accapezzato, D. , R. Caccavale , M. P. Paroli , et al. 2023. “Advances in the Pathogenesis and Treatment of Systemic Lupus Erythematosus.” International Journal of Molecular Sciences 24, no. 7: 6578. 37047548 10.3390/ijms24076578 PMC10095030

[brb370754-bib-0002] Barrett, T. , S. E. Wilhite , P. Ledoux , et al. 2013. “NCBI GEO: Archive for Functional Genomics Data Sets–Update.” Nucleic Acids Research 41: D991–D995. 23193258 10.1093/nar/gks1193 PMC3531084

[brb370754-bib-0003] Carter, P. , S. Yuan , S. Kar , et al. 2022. “Coffee Consumption and Cancer Risk: A Mendelian Randomisation Study.” Clinical Nutrition 41, no. 10: 2113–2123. 36067583 10.1016/j.clnu.2022.08.019 PMC7613623

[brb370754-bib-0004] Didelez, V. , and N. Sheehan . 2007. “Mendelian Randomization as an Instrumental Variable Approach to Causal Inference.” Statistical Methods in Medical Research 16, no. 4: 309–330. 17715159 10.1177/0962280206077743

[brb370754-bib-0005] Fang, P. , X. Liu , Y. Qiu , et al. 2023. “Exploring Causal Correlations Between Inflammatory Cytokines and Ankylosing Spondylitis: A Bidirectional Mendelian‐Randomization Study.” Frontiers in immunology 14: 1285106. 38054001 10.3389/fimmu.2023.1285106 PMC10694192

[brb370754-bib-0006] Gupta, V. , G. K. Walia , and M. P. Sachdeva . 2017. “Mendelian Randomization': An Approach for Exploring Causal Relations in Epidemiology.” Public Health 145: 113–119. 28359378 10.1016/j.puhe.2016.12.033

[brb370754-bib-0007] Hengrui, L. 2023. “An Example of Toxic Medicine Used in Traditional Chinese Medicine for Cancer Treatment.” Journal of Traditional Chinese Medicine 43, no. 2: 209–210. 36994507 10.19852/j.cnki.jtcm.2023.02.001 PMC10012187

[brb370754-bib-0008] Henriques, A. , L. Teixeira , L. Inês , et al. 2013. “NK Cells Dysfunction in Systemic Lupus Erythematosus: Relation to Disease Activity.” Clinical Rheumatology 32, no. 6: 805–813. 23377197 10.1007/s10067-013-2176-8

[brb370754-bib-0009] Herrada, A. A. , N. Escobedo , M. Iruretagoyena , et al. 2019. “Innate Immune Cells' Contribution to Systemic Lupus Erythematosus.” Frontiers in Immunology 10: 772. 31037070 10.3389/fimmu.2019.00772 PMC6476281

[brb370754-bib-0010] Higbee, D. H. , R. Granell , E. Sanderson , G. Davey Smith , and J. W. Dodd . 2021. “Lung Function and Cardiovascular Disease: A Two‐Sample Mendelian Randomisation Study.” European Respiratory Journal 58, no. 3: 2003196. 33574079 10.1183/13993003.03196-2020

[brb370754-bib-0011] Hu, J. , and S. Szymczak . 2023. “A Review On Longitudinal Data Analysis With Random Forest.” Briefings in Bioinformatics 24, no. 2: bbad002. 36653905 10.1093/bib/bbad002 PMC10025446

[brb370754-bib-0012] Ichinose, K. , C. M. Hedrich , V. R. Moulton , and M. Mizui . 2021. “Editorial: Focusing on T‐Cells for Novel Treatments of Systemic Lupus Erythematosus.” Frontiers in Immunology 12: p. 744866. 34421933 10.3389/fimmu.2021.744866 PMC8377347

[brb370754-bib-0013] Jiménez, S. , R. Cervera , J. Font , and M. Ingelmo . 2003. “The Epidemiology of Systemic Lupus Erythematosus.” Clinical Reviews in Allergy & Immunology 25, no. 1: p. 3–12. 12794256 10.1385/CRIAI:25:1:3

[brb370754-bib-0014] Joshi, A. S. , B. Nebenfuehr , V. Choudhary , et al. 2018. “Lipid Droplet and Peroxisome Biogenesis Occur at the Same ER Subdomains.” Nature Communications 9, no. 1: p. 2940. 10.1038/s41467-018-05277-3 PMC6063926 30054481

[brb370754-bib-0015] Kang, J. , Y. J. Choi , I. Kim , et al. 2021. “LASSO‐Based Machine Learning Algorithm for Prediction of Lymph Node Metastasis in T1 Colorectal Cancer.” Cancer Research and Treatment: Official Journal of Korean Cancer Association 53, no. 3: p. 773–783. 10.4143/crt.2020.974 PMC8291173 33421980

[brb370754-bib-0016] Kato, H. , and J. M. Kahlenberg . 2024. “Emerging Biologic Therapies for Systemic Lupus Erythematosus.” Current Opinion in Rheumatology 36, no. 3: p. 169–175. 38299618 10.1097/BOR.0000000000001003

[brb370754-bib-0017] Kiriakidou, M. , and C. L. Ching . 2020. “Systemic Lupus Erythematosus.” Annals of Internal Medicine 172, no. 11: p. ITC81–ITC96. 32479157 10.7326/AITC202006020

[brb370754-bib-0018] Kucuksezer, U. C. , E. Aktas Cetin , F. Esen , et al. 2021. “The Role of Natural Killer Cells in Autoimmune Diseases.” Frontiers in Immunology 12: p. 622306. 33717125 10.3389/fimmu.2021.622306 PMC7947192

[brb370754-bib-0019] Leek, J. T. , W. E. Johnson , H. S. Parker , A. E. Jaffe , and J. D. Storey . 2012. “The sva Package for Removing Batch Effects and Other Unwanted Variation in High‐Throughput Experiments.” Bioinformatics 28, no. 6: 882–883. 22257669 10.1093/bioinformatics/bts034 PMC3307112

[brb370754-bib-0020] Li, Q. , H. Liu , J. Jin , et al. 2025. “Traditional Chinese Medicine Properties and Microcalorimetry: BioScience Evaluation.” Med Research 70002, no. 2: 103–121.

[brb370754-bib-0021] Li, Y. , and H. Liu . 2022. “Clinical Powers of Aminoacyl tRNA Synthetase Complex Interacting Multifunctional Protein 1 (AIMP1) for Head‐Neck Squamous Cell Carcinoma.” Cancer Biomarkers 34: 359–374. 35068446 10.3233/CBM-210340 PMC12364190

[brb370754-bib-0022] Liu, H. 2020. “Effect of Traditional Medicine on Clinical Cancer.” Biomedical Journal of Scientific & Technical Research 30, no. 4: p. 23548–23551.

[brb370754-bib-0023] Liu, H. 2022. “Toxic Medicine Used in Traditional Chinese Medicine for Cancer Treatment: Are Ion Channels Involved?” Journal of Traditional Chinese Medicine 42, no. 6: 1019–1022. 36378062 10.19852/j.cnki.jtcm.20220815.005 PMC9924727

[brb370754-bib-0024] Liu, H. 2023. “Association Between Sleep Duration and Depression: A Mendelian Randomization Analysis.” Journal of Affective Disorders 335: 152–154. 37178827 10.1016/j.jad.2023.05.020

[brb370754-bib-0025] Liu, H. 2025. “Role of Traditional Chinese Medicine in Supporting Liver Transplantation Outcomes.” World Journal of Transplantation 15, no. 3: 103904. 40881752 10.5500/wjt.v15.i3.103904 PMC12038594

[brb370754-bib-0026] Liu, H. , Z. Guo , and P. Wang . 2024. “Genetic Expression in Cancer Research: Challenges and Complexity.” Gene Reports 37: 102042.

[brb370754-bib-0027] Liu, H. , M. Karsidag , K. Chhatwal , P. Wang , and T. Tang . 2025. “Single‐Cell and Bulk RNA Sequencing Analysis Reveals CENPA as a Potential Biomarker and Therapeutic Target in Cancers.” PLoS ONE 20, no. 1: p. e0314745. 39820192 10.1371/journal.pone.0314745 PMC11737691

[brb370754-bib-0028] Liu, H. , and Y. Li . 2022. “Potential Roles of Cornichon Family AMPA Receptor Auxiliary Protein 4 (CNIH4) in Head and Neck Squamous Cell Carcinoma.” Cancer Biomarkers 35, no. 4: p. 439–450. 36404537 10.3233/CBM-220143 PMC12364253

[brb370754-bib-0029] Liu, H. , Y. Li , M. Karsidag , T. Tu , and P. Wang . 2025. “Technical and Biological Biases in Bulk Transcriptomic Data Mining for Cancer Research.” Journal of Cancer 16, no. 1: p. 34–43. 39744578 10.7150/jca.100922 PMC11660120

[brb370754-bib-0030] Liu, H. , and T. Tang . 2023a. “A Bioinformatic Study of IGFBPs in Glioma Regarding Their Diagnostic, Prognostic, and Therapeutic Prediction Value.” American Journal of Translational Research 15, no. 3: p. 2140–2155. 37056850 PMC10086936

[brb370754-bib-0031] Liu, H. , and T. Tang . 2023b. “MAPK Signaling Pathway‐Based Glioma Subtypes, Machine‐Learning Risk Model, and Key Hub Proteins Identification.” Scientific Reports 13, no. 1: p. 19055. 37925483 10.1038/s41598-023-45774-0 PMC10625624

[brb370754-bib-0032] Liu, H. , and J. Weng . 2022a. “A Pan‐Cancer Bioinformatic Analysis of RAD51 Regarding the Values for Diagnosis, Prognosis, and Therapeutic Prediction.” Frontiers in Oncology 12: 858756. 35359409 10.3389/fonc.2022.858756 PMC8960930

[brb370754-bib-0033] Liu, H. , and J. Weng . 2022b. “A Comprehensive Bioinformatic Analysis of Cyclin‐Dependent Kinase 2 (CDK2) in Glioma.” Gene 822: p. 146325. 35183683 10.1016/j.gene.2022.146325

[brb370754-bib-0034] Liu, H. , R. Xie , Q. Dai , J. Fang , Y. Xu , and B. Li,. 2023. “Exploring the Mechanism Underlying Hyperuricemia Using Comprehensive Research On Multi‐Omics.” Scientific Reports 13, no. 1: p. 7161. 37138053 10.1038/s41598-023-34426-y PMC10156710

[brb370754-bib-0035] Lood, C. , S. Arve , J. Ledbetter , and K. B. Elkon . 2017. “TLR7/8 Activation in Neutrophils Impairs Immune Complex Phagocytosis Through Shedding of FcgRIIA.” Journal of Experimental Medicine 214, no. 7: p. 2103–2119. 28606989 10.1084/jem.20161512 PMC5502427

[brb370754-bib-0036] Melki, I. , I. Allaeys , N. Tessandier , et al. 2020. “FcγRIIA Expression Accelerates Nephritis and Increases Platelet Activation in Systemic Lupus Erythematosus.” Blood 136, no. 25: p. 2933–2945. 33331924 10.1182/blood.2020004974 PMC7751357

[brb370754-bib-0037] Mills, J. A. 1994. “Systemic Lupus Erythematosus.” New England Journal of Medicine 330, no. 26: p. 1871–1879. 8196732 10.1056/NEJM199406303302608

[brb370754-bib-0038] Möckel, T. , F. Basta , J. Weinmann‐Menke , and A. Schwarting . 2021. “B Cell Activating Factor (BAFF): Structure, Functions, Autoimmunity and Clinical Implications in Systemic Lupus Erythematosus (SLE).” Autoimmunity Reviews 20, no. 2: p. 102736. 33333233 10.1016/j.autrev.2020.102736

[brb370754-bib-0039] Nagelkerke, S. Q. , D. E. Schmidt , M. De Haas , and T. W. Kuijpers . 2019. “Genetic Variation in Low‐To‐Medium‐Affinity Fcγ Receptors: Functional Consequences, Disease Associations, and Opportunities for Personalized Medicine.” Frontiers in Immunology 10: 2237. 31632391 10.3389/fimmu.2019.02237 PMC6786274

[brb370754-bib-0040] Nehar‐Belaid, D. , S. Hong , R. Marches , et al. 2020. “Mapping Systemic Lupus Erythematosus Heterogeneity at the Single‐Cell Level.” Nature Immunology 21, no. 9: p. 1094–1106. 32747814 10.1038/s41590-020-0743-0 PMC7442743

[brb370754-bib-0041] Ou, L. , Z. Zhu , Y. Hao , et al. 2024. “1,3,6‐Trigalloylglucose: A Novel Potent Anti‐*Helicobacter pylori* Adhesion Agent Derived From Aqueous Extracts of *Terminalia chebula* Retz.” Molecules 29, no. 5: p. 1161. 38474673 10.3390/molecules29051161 PMC10935070

[brb370754-bib-0042] Peng, C. , Z. Feng , L. Ou , et al. 2023. “ *Syzygium aromaticum* Enhances Innate Immunity by Triggering Macrophage M1 Polarization and Alleviates *Helicobacter pylori*‐Induced Inflammation.” Journal of Functional Foods 107: p. 105626.

[brb370754-bib-0043] Sharabi, A. , and G. C. Tsokos . 2020. “T Cell Metabolism: New Insights in Systemic Lupus Erythematosus Pathogenesis and Therapy.” Nature Reviews Rheumatology 16, no. 2: p. 100–112. 31949287 10.1038/s41584-019-0356-x

[brb370754-bib-0044] Siegel, M. , and S. L. Lee . 1973. “The Epidemiology of Systemic Lupus Erythematosus.” Seminars in Arthritis and Rheumatism 3, no. 1: p. 1–54. 4125999 10.1016/0049-0172(73)90034-6

[brb370754-bib-0045] Subramanian, A. , P. Tamayo , V. K. Mootha , et al. 2005. “Gene Set Enrichment Analysis: A Knowledge‐Based Approach for Interpreting Genome‐Wide Expression Profiles.” PNAS 102, no. 43: 15545–15550. 16199517 10.1073/pnas.0506580102 PMC1239896

[brb370754-bib-0047] Thomas, G. , R. Tacke , C. C. Hedrick , and R. N. Hanna . 2015. “Nonclassical Patrolling Monocyte Function in the Vasculature.” Arteriosclerosis, Thrombosis, and Vascular Biology 35, no. 6: 1306–1316. 25838429 10.1161/ATVBAHA.114.304650 PMC4441550

[brb370754-bib-0048] Tsang‐A‐Sjoe, M. W. P. , S. Q. Nagelkerke , I. E. M. Bultink , et al. 2016. “Fc‐Gamma Receptor Polymorphisms Differentially Influence Susceptibility to Systemic Lupus Erythematosus and Lupus Nephritis.” Rheumatology 55, no. 5: p. 939–948. 26748351 10.1093/rheumatology/kev433

[brb370754-bib-0049] Vyse, T. J. , and B. L. Kotzin . 1998. “Genetic Susceptibility to Systemic Lupus Erythematosus.” Annual Review of Immunology 16: 261–292. 10.1146/annurev.immunol.16.1.261 9597131

[brb370754-bib-0050] Wang, C. , G. Chen , J. Wang , et al. 2016. “Effect of Herba Epimedium Extract on Bone Mineral Density and Microstructure in Ovariectomised Rat.” Journal of Pharmaceutical and Biomedical Sciences 6, no. 5: 275–278.

[brb370754-bib-0051] Wang, H. , S. Mo , L. Yang , et al. 2020. “Effectiveness Associated With Different Therapies for Senile Osteopo‐Rosis: a Network Meta‐Analysis.” Journal of Traditional Chinese Medicine 40, no. 1: p. 17–27. 32227762

[brb370754-bib-0052] Wang, Y. , Z. Huang , Y. Xiao , W. Wan , and X. Yang . 2022. “The Shared Biomarkers and Pathways of Systemic Lupus Erythematosus and Metabolic Syndrome Analyzed by Bioinformatics Combining Machine Learning Algorithm and Single‐Cell Sequencing Analysis.” Frontiers in Immunology 13: p. 1015882. 36341378 10.3389/fimmu.2022.1015882 PMC9627509

[brb370754-bib-0053] Xiong, H. , M. Cui , N. Kong , et al. 2023. “Cytotoxic CD161(‐)CD8(+) T(EMRA) Cells Contribute to the Pathogenesis of Systemic Lupus Erythematosus.” EBioMedicine 90: p. 104507. 36893588 10.1016/j.ebiom.2023.104507 PMC10011749

[brb370754-bib-0054] Xu, Z. , A. M. Rasteh , A. Dong , P. Wang , and H. Liu . 2024. “Identification of Molecular Targets of Hypericum Perforatum in Blood for Major Depressive Disorder: A Machine‐Learning Pharmacological Study.” Chinese Medicine 19, no. 1: p. 141. 39385284 10.1186/s13020-024-01018-5 PMC11465934

[brb370754-bib-0055] Yang, J. , X. Yang , L. Wang , and M. Li . 2020. “B Cells Control Lupus Autoimmunity by Inhibiting Th17 and Promoting Th22 Cells.” Cell Death & Disease 11, no. 3: p. 164. 32127533 10.1038/s41419-020-2362-y PMC7054432

[brb370754-bib-0056] Zhao, M. , F. Wei , H. Li , et al. 2023. “Serum Vitamin D Levels and Sjogren's Syndrome: Bi‐Directional Mendelian Randomization Analysis.” Arthritis Research & Therapy 25, no. 1: p. 79. 37189174 10.1186/s13075-023-03062-2 PMC10184420

[brb370754-bib-0057] Zoccali, C. 2017. “The Challenge of Mendelian Randomization Approach.” Current Medical Research and Opinion 33, no. S3: p. 5–8. 10.1080/03007995.2017.1378514 28952387

