# Supplementary material for: Mendelian Randomization and Machine Learning Reveal Immune Cell and Gene Drivers in Systemic Lupus Erythematosus
Source: Brain Behav. 2025 Sep 16;15(9):e70754. doi: 10.1002/brb3.70754 (PMC12441009; doi:10.1002/brb3.70754)
Supplement: Supplementary file 1 — Supporting Table 1: SLE‐related microarray datasets. [file BRB3-15-e70754-s001.docx]

Supplementary Table 1. SLE-related microarray datasets.

| Dataset | Platform | Total | SLE | Normal | Samples |
| --- | --- | --- | --- | --- | --- |
| GSE50772 | GPL570 | 81 | 61 | 20 | Blood |
| GSE81622 | GPL10558 | 40 | 15 | 25 | Blood |
| GSE61635 | GPL570 | 129 | 99 | 30 | Blood |
