# Supplementary material for: Mendelian Randomization and Machine Learning Reveal Immune Cell and Gene Drivers in Systemic Lupus Erythematosus
Source: Brain Behav. 2025 Sep 16;15(9):e70754. doi: 10.1002/brb3.70754 (PMC12441009; doi:10.1002/brb3.70754)
Supplement: Supplementary file 6 — Supporting Table 6:| Gene name conversion. [file BRB3-15-e70754-s003.docx]

Supplementary Table 6| Gene name conversion.

| Gene description | Gene symbol | Type |
| --- | --- | --- |
| 1-Acylglycerol-3-phosphate O-acyltransferase 4 | AGPAT4 | DEAG |
| BCAR3 adaptor protein, NSP family member | BCAR3 | DEAG |
| CD4 molecule | CD4 | DEAG |
| Discoidin domain receptor tyrosine kinase 2 | DDR2 | DEAG |
| F11 receptor | F11R | DEAG |
| FERM, ARH/RhoGEF, and pleckstrin domain protein 1 | FARP1 | DEAG |
| Fc gamma receptor IIa | FCGR2A | DEAG |
| FERM and PDZ domain containing 1 | FRMPD1 | DEAG |
| HECT, C2 and WW domain containing E3 ubiquitin protein ligase 2 | HECW2 | DEAG |
| Integrin subunit alpha 11 | ITGA11 | DEAG |
| Multiple C2 and transmembrane domains containing 2 | MCTP2 | DEAG |
| NudC domain containing 3 | NUDCD3 | DEAG |
| Peptidylprolyl isomerase F | PPIF | DEAG |
| RAS p21 protein activator 3 | RASA3 | DEAG |
| Sulfatase modifying factor 1 | SUMF1 | DEAG |
| Transmembrane protein 181 | TMEM181 | DEAG |
| Ubiquitin-specific peptidase 8 | USP8 | DEAG |
